# Hand Hygiene Auditing: Is It a Roadway to Improve Adherence to Hand Hygiene Among Hospital Personnel?

**DOI:** 10.7759/cureus.25221

**Published:** 2022-05-22

**Authors:** Vithiya Ganesan, Raja Sundaramurthy, Rajendran Thiruvanamalai, Monica Raghavan, Sunil Kumar D Chavan, Rajeshwari Pusa, Varatharajan Sakthivadivel, Archana Gaur, Yuvaraj Balan

**Affiliations:** 1 Department of Microbiology, Velammal Medical College Hospital and Research Institute, Madurai, IND; 2 Department of Microbiology, All India Institute of Medical Sciences, Bibinagar, Hyderabad, IND; 3 Department of Internal Medicine, All India Institute of Medical Sciences, Bibinagar, Hyderabad, IND; 4 Department of Physiology, All India Institute of Medical Sciences, Bibinagar, Hyderabad, IND; 5 Department of Biochemistry, All India Institute of Medical Sciences, Bibinagar, Hyderabad, IND

**Keywords:** hh surveillance, hai prevention, hand hygiene audit, behavioral change, hand hygiene compliance

## Abstract

Background

Over the years, there has been an increase in hospital-acquired infections (HAIs) among patients in India. One of the main reasons is a lack of compliance with infection control guidelines, such as hand hygiene. So the present study was conducted to determine the compliance of hand hygiene among healthcare workers in a private tertiary care teaching hospital in South India.

Materials and methods

The prospective observational study was carried out between April 2017 and March 2020. Nineteen areas were directly observed for hand hygiene (HH) compliance. At each location, HH audit was conducted for one hour per day for five days per month. HH complete adherence rate (HHCAR) and HH partial adherence rate (HHPAR) were analyzed.

Results

Nine hundred and twenty observation periods were completed during the entire study period. Overall, hand hygiene complete adherence rate was 29.9% (11,981/39,998); partial adherence rate was 45.3% (18,131/39,998) and the non-adherence rate was 24.7% (9886/39,998). A better adherence rate was seen among nurses (44.7%), followed by other staff (33.7%) and doctors (33.04%). Moment-specific adherence rates show almost equal adherence rates of 50.7%, 50.75%, and 50.1%, respectively, for moments 2, 3, and 4, and comparatively low for moments 1 and 5 (48.4% and 47.6%, respectively).

Conclusion

Despite adequate hand hygiene facilities, compliance remains low. Hand hygiene is a bundle care approach that needs to consider factors including healthcare staff, clinical, institutional, environmental, and behavioral changes. Multimodal interventions and multidisciplinary commitment are mandatory for sustained compliance.

## Introduction

The hospital sector in India is growing rapidly, with the private sector having more infrastructure than the public sector. Obviously, better standards and reliable service operations are expected from these organizations. This mandates many private hospitals to enroll in accreditation programs. The hospital infection control chapter is one of the important hospital standards. Over the years, there has been an increase in hospital-acquired infections (HAI) among patients in India. One of the main reasons is a lack of compliance with infection control guidelines, such as hand hygiene. HAIs are the major cause of morbidity and mortality in healthcare settings throughout the world, contributing to 7-10% of hospital admissions [1-3].

Many factors like lack of knowledge among healthcare workers (HCWs) regarding the importance of hand hygiene in preventing disease transmission, incorrect technique, poor access to handwashing facilities, lack of motivation, understaffing, and irritant contact dermatitis contribute to poor adherence to hand hygiene [4-7]. The goal of every healthcare institution will be to improve adherence to hand hygiene to 100% and to bring the HAI rate as low as possible.

Monitoring the hand hygiene (HH) compliance will be the fundamental quality indicator in all healthcare settings. The monitoring of HH can be performed by direct observations of HH practices, measuring the product use, conducting a survey, and, more recently, through video monitoring and electronic surveillance [8].

Direct observation is still considered the gold standard method when compared to other modern methods available as it will also give other information like volume and duration of HH products used, compliance to various steps of HH techniques, and method of drying. Also, it is the most feasible methodology in the limited-resource setting, which will give information regarding all elements of HH [8,9].

Hand hygiene is an important and effective measure in the prevention of healthcare-associated infections. Hand hygiene compliance was one of the quality indicators of the hospital infection control department when the organization applied for the accreditation process along with written protocols, posters at strategic locations, conveniently located functional sinks with elbow operated taps, an uninterrupted water supply, availability of liquid handwash, and paper towels [9]. In this context, we planned to conduct a surveillance of adherence to hand hygiene practices among hospital personnel.

This article was previously presented as an abstract at the 13th International Symposium on Antimicrobial Agents and Resistance (ISAAR) Virtual Congress, September 9-10, 2021, Volume 58/3 PHI-001.

## Materials and methods

The study was conducted in a private tertiary care medical college hospital with a super specialty having 1500 beds and 14 intensive care units (ICUs) catering to the needs of many neighboring districts. The present study was conducted while our hospital was preparing to undergo National Accreditation Board for Hospitals and Healthcare Providers (NABH) assessment. The incidence of device-associated infection was very high, especially catheter-associated urinary tract infection (CAUTI),** **as high as 10.7 per 1000 catheter days and ventilator-associated pneumonia (VAP) 15 per 1000 ventilator days, which also mandates the hand hygiene audit in our setup [10].

The prospective observational study was carried out over a period of 36 months between April 2017 and March 2020 after obtaining the Institutional Ethics Committee approval (IEC No. VMCIEC/49). During the early months of the study, only five adult ICUs were audited due to constraints in the availability of dedicated infection control nurses (ICNs). Realizing the important role of hand hygiene as a measure of quality and patient safety, the organization supported the program with infection control nurses adequate enough to conduct audits in all the critical areas. By the end of the study, 16 (14 ICUs and two postoperative wards) areas were directly observed for HH compliance. At each of the locations, the HH audit was conducted for one hour/day for five days/month. Thus, in total, there were 920 observation periods (each conducted for one hour), and 22,600 minutes of observation were completed during the entire study period. We evaluated the level of compliance across different units and among different categories of HCWs. The HH audit form used in our study was designed based on the World Health Organization (WHO) HH audit tool kit.

The auditors were trained infection control nurses. Immense efforts were taken to reduce all the possible biases expected to rise during the audit process and to ensure standardization and reliability of the audit. The auditors were trained prior to the audit to reduce inter-auditor variation in data collection. Special emphasis was given to addressing the double-counting, which is the most common inter-auditor bias in HH audits. The auditors were conducting the HH audit simultaneously along with their other routine work (e.g., HAI surveillance work, biomedical waste audit, environmental cleaning audit, care bundle audit, etc.), so the HCWs were not aware that their HH practice was being monitored; thus minimizing the observational bias (i.e., Hawthorne effect). Also, we have done a month-wise rotation of the observers to minimize confirmation bias.

The audit was carried out on a random schedule, thus obviating the confounding bias of work pressure influencing the HH compliance.

HH audit was carried out as direct observation and filling the HH audit form based on a standardized WHO method for HH moments (five moments - before touching a patient, before an aseptic procedure, after body fluid exposure risk, after touching a patient, and after touching patient surroundings) and HH techniques (seven steps - palm to palm, back to palm with finger interlacing, palm to palm with finger interlacing, interlocking, rotation movement of the thumb, the tip of the finger in the opposite palm, rubbing wrist with opposite hand) by a trained infection control nurses.

Parameters collected during the HH audit

The auditors recorded the following information: date and time of the audit, the profession of the healthcare workers, HH opportunities (HH moments) available, the duration for which the HH is performed, and the steps of HH followed. However, the name of the HCWs was not recorded. The HH event was marked as "completely followed" when all the seven WHO steps of HH were performed for the recommended duration (>20 seconds for hand rub and >40 seconds for hand wash). The auditing included doctors (intensivists, visiting consultants, interns) nurses, physiotherapists, and housekeeping personnel.

Interventions implemented

Onsite advice and corrections were given to the HCWs at the end of the observation period on a daily basis so as to improve the HH practices subsequently. The gradual improvement in the hand hygiene compliance of the HCWs in the same as well as subsequent posting was evaluated.

Statistical plan and data analysis

Table 1 depicts the list of dependable variables and the formulae used for their calculation. The HH complete adherence rate (HHCAR), profession-specific HHCAR (e.g., doctors, nurses, and others), and moment-specific HHCAR (for each WHO moment) were calculated. The impact of conducting the HH audit was assessed by observing the trend analysis of the month-wise HHCAR. Data was entered in the prescribed format in Excel and validated by the hospital infection control officer. The monthly HH audit report was shared with the ICU and/or presented in the hospital infection control committee (HICC) meetings.

**Table 1:** List of dependent variables and the formulae HH - hand hygiene

Dependable variables	Formulae
Hand hygiene complete adherence rate (HHCAR)	[Number of times HH completely performed (all steps and appropriate duration) / total opportunities observed] X 100
Profession-specific hand hygiene adherence rate	[HH performed by each profession / total opportunities observed] X 100
Moment-specific hand hygiene adherence rate	[HH performed for each WHO moment (opportunity) / total moments (opportunities) observed] X 100

## Results

There were 920 observation periods (each conducted for one hour), and 22,600 minutes of observation were completed during the entire study period. As shown in Table 2, a total of 39,998 hand hygiene moments were available during the entire study period. Overall hand hygiene complete adherence rate was 29.9% (11,981/39,998). The trend of hand hygiene compliance over the study period was non-monotonic, with ups and downs. In the present study, though there was a gradual increase in adherence rate during the early phase of the study, from April 2017 (34.8%) to March 2018 (63.8%), but the rate was not sustained thereafter. There was a consistent fall in adherence rate after April 2019 for the next six months, going down to 22.5% in October 2019, followed by a gradual increase in rate.

**Table 2:** Month-wise hand hygiene complete adherence rate (HHCAR) HH - hand hygiene

Month	HH moments available	HH completely followed	HH complete adherence rate (HHCAR %)
April 2017	1157	403	34.8
May 2017	1141	442	38.7
June 2017	1169	522	44.7
July 2017	1265	564	44.6
August 2017	1231	544	44.2
September 2017	1228	610	49.7
October 2017	1348	689	51.1
November 2017	1131	591	52.3
December 2017	1161	644	55.5
January 2018	1291	748	57.9
February 2018	1323	819	61.9
March 2018	1327	842	63.5
July 2018	1788	468	52.5
September 2018	1015	455	41.4
October 2018	294	85	57.4
November 2018	128	72	24.2
January 2019	1308	605	42.2
February 2019	425	164	45.6
April 2019	3114	625	55.3
May 2019	1035	441	27.2
July 2019	1723	866	22.9
August 2019	716	464	29.7
September 2019	2423	1172	25.9
October 2019	1219	551	22.5
November 2019	2985	1445	22.8
December 2019	1483	690	23.3
January 2020	2175	981	27.0
February 2020	2427	1220	35.9
March 2020	968	409	45.1

Table 3 represents the location-wise hand hygiene complete adherence rate. Each ICU had a single secured entrance with alcohol-based hand rub dispensers available. One alcohol-based hand rub dispenser for every bed within each unit. Hand hygiene posters were available at appropriate sites. All nursing, housekeeping staff, and allied health staff received basic infection control training as a continuous process and an induction course for all new recruits. But patients in different types of ICUs had different requirements of care, resulting in differing hand hygiene opportunities. Nurse patient ratio and staff attrition rate were different among ICUs. Adherence rates ranged from 15.9% in high dependency units to 46.9% in neonatal ICU.

**Table 3:** Hand hygiene compliance among personnel by setting HHCAR - hand hygiene complete adherence rate; ICU - intensive care unit

Location	Moments available	Completely followed	HHCAR
Surgical ICU	5530	1320	23.8
Medical ICU	5429	1422	26.1
Specialty ICU	4362	1129	25.8
Neuro ICU	4567	1277	27.9
Cardiothoracic ICU	4999	1633	32.6
Neonatal ICU	2364	1111	46.9
Pediatric ICU	2378	909	38.2
Postoperative ward	1547	715	46.2
Specialty surgical ICU	1323	376	28.4
Orthopedic postoperative ward	640	231	36
High dependent Unit	695	111	15.9
Liver ICU	1225	326	26.6
Intensive cardiac care unit	1708	438	25.6
Trauma ICU	449	203	45.2
Specialty neurotrauma ICU	1767	444	25.1
Cardiac ICU	1015	248	24.4

Table 4 represents the profession-specific hand hygiene adherence rate. The highest adherence rate was seen among nurses (47.3%), followed by housekeepers (28.6%) and physiotherapists (21.3%). Among doctors, decreasing order of adherence is as follows - intensivists (12.08%), interns (10.3%), and visiting consultants (3.4%).

**Table 4:** Profession-specific hand hygiene adherence rate HH - hand hygiene;  HHCAR - hand hygiene complete adherence rate

Profession	HH moments available	HH completely followed	HHCAR %
Intensivists	12628	1525	12.1
Visiting consultants	1209	41	3.4
Interns	481	49	10.3
Physiotherapists	535	114	21.3
Nurses	21347	10097	47.3
Housekeepers	3798	1086	28.6

Table 5 shows moment-specific adherence rates showing almost equal adherence rates of 50.7%, 50.75%, and 50.1% for moments 2, 3, and 4, respectively. Adherence rate was comparatively low for moments 1 and 5, i.e., 48.4% and 47.6%, respectively.

**Table 5:** Moment-specific adherence rate HH - hand hygiene;  HHCAR - hand hygiene complete adherence rate

Moment	HH moments available	HH completely followed	HHCAR %
Moment 1	9559	4626	48.4
Moment 2	7399	3751	50.7
Moment 3	7559	3832	50.7
Moment 4	8319	4167	50.1
Moment 5	6999	3331	47.6

## Discussion

In the present study, though there was a gradual increase in adherence rate during the early phase of the study (from April 2017 to March 2018), the rate was not sustained thereafter. There was a consistent fall in adherence rate after April 2019 for the next six months, followed by a gradual increase in rate. This implies that HH studies with such a large duration of observation period are critical to producing more accurate and reliable data on HH compliance. Even after long periods of intensive education and training programs, there was a progressive decline in the HH compliance rate. A number of investigators have reported improved adherence after implementing various interventions, but most studies had short follow-up periods and did not establish if improvements were of long duration. Few studies have reported sustained improvement as a consequence of the long-running implementation of programs aimed at promoting optimal adherence to hand hygiene policies [11,12].

At the institutional level, factors like availability of written protocols, posters at strategic locations, ample supply of alcohol-based hand rub placed at bedside, conveniently located functional sinks with elbow operated taps, uninterrupted water supply, liquid handwash, and paper towels were prioritized. Some of the factors not addressed at the institutional level include administrative sanctions for noncompliance and a high attrition rate of staff [13]. Organizations that promote hand hygiene should consider all these variables. Other factors which could be potential reasons for reduced compliance are as follows. In the present study, compliance rates were communicated consistently, but the feedback was not obtained from locations with low adherence rates. This could have been critical to keeping everyone engaged and aware of the issue. Some of the issues like understaffing, overcrowding of patients, and extended working shifts would have been rationally sorted out.

At the individual level, staff are adequately knowledged but putting it to practice is an issue. Adequacy of staff knowledge was assessed during the regular visits by infection control nurses by means of staff interviews. In the present study, although some HCWs knew that they were under observation, overall compliance of HH continued to be very low. Even though the staff was aware of the low HH compliance rate, they did not feel an urgency to improve adherence because the consequences were not immediately obvious. To inculcate behavioral changes, hand hygiene compliance can be incorporated into the measure of an employee's overall performance.

Sustained adherence to hand hygiene remains a challenge. The question of how to change a healthcare worker's attitude needs to be addressed. Some of the tools, such as rewards for compliance, were tested in our institute, and the results were promising. Hand hygiene awareness week was observed in the middle of October 2019 when the overall compliance rate was just 22.5%, and trophies were awarded to the location and healthcare workers having the highest adherence rate. Such appreciation helped in changing their practices, and a gradual rise in compliance was observed.

Also, the results showed highly variable levels of adherence to the best hygiene practices in all the ICUs involved in the study, with compliance rates ranging from 23.8% in surgical ICU (understaffing and overcrowding of patients) to 46.9% in neonatal ICU (appropriate staffing and low attrition rate). The difference could also be attributed to more moments available in surgical ICU (5530 opportunities) compared to neonatal ICU (2364 opportunities). Pittet et al. had shown that settings with a very high number of HH opportunities (> 60/h of patient care) would have generally poor compliance rates compared to low (from 0 to 20 HH opportunities) [1].

Furthermore, we found differences in the levels of adherence to the HH practices among HCWs. In particular, doctors had lower compliance rates than other HCWs. This is in accordance with most studies in the literature, including a meta-analysis [14]. We observed that the compliance with moment 1 (before touching patients), as well as moment 5 (after touching patients' environment), was low compared to other moments. They tend to practice HH more when they are at risk of contracting microorganisms from patients but ignore HH practice when patients are at risk of contracting an infection.

In the present study, doctors were not oriented to basic infection control measures, and no induction course was offered for any of the newly employed. Most of the senior doctors never had any sensitization to hand hygiene practices.

Some of them were skeptical regarding the value of hand hygiene [15]. Some of them were resistant to change or considered it a threat to their autonomy. Senior visiting consultants got offended when they were verbally reminded by junior intensivists. Among doctors, decreasing order of compliance is as follows - intensivists, interns, and visiting consultants. One way that can be tried is to empower and encourage patients to challenge non-compliant staff to increase adherence to appropriate hand hygiene.

Moment-specific adherence rates showed an almost equal adherence rate of 50.7%, 50.75, and 50.1 % for moments 2, 3, and 4, respectively. Adherence rate was comparatively low for moments 1 and 5, i.e., 48.4% and 47.6%, respectively. This pattern is similar to other studies, including a meta-analysis that found lower compliance rates before patient contact (21%) compared to after patient contact (47%) [16].

Limitations of the study

Shortcomings of direct observation are well known. Apart from the well-known Hawthorne effect, employing individuals for manual observation is labor-intensive, costly and for large organizations which aim to permanently improve hand hygiene, a small army of auditors will be required to patrol the areas at all times. In the current study, we could conduct audits only in ICUs and two postoperative wards with the available four trained infection control nurses, which was practically difficult. Also, the audit was done only during the daytime. So diurnal variation was not studied. Another potential limitation to consider could be that the study was conducted in a single site, limiting its generalizability.

## Conclusions

Despite being an institutional priority in the context of the impending final accreditation process, hand hygiene compliance remains low. It can be attributed to the high staff attrition rate, lack of administrative sanctions for noncompliance, and attitude of healthcare personnel. HH auditing and reporting to the individual level, appreciation in terms of the trophy has helped in our setup for changing the HCWs practices and gradual rise in compliance. Multimodal interventions and multidisciplinary commitment are mandatory for sustained compliance.
